# A Genome-Wide Association Study of Female Sexual Dysfunction

**DOI:** 10.1371/journal.pone.0035041

**Published:** 2012-04-11

**Authors:** Andrea Burri, Pirro Hysi, Alex Clop, Qazi Rahman, Tim D. Spector

**Affiliations:** 1 Biological and Experimental Psychology Group, School of Biological and Chemical Sciences, Queen Mary University of London, London, United Kingdom; 2 Department of Twin Research and Genetic Epidemiology, King’s College London, London, United Kingdom; 3 Department of Medical and Molecular Genetics, School of Medicine, King's College London, London, United Kingdom; Central China Normal University, China

## Abstract

**Background:**

Female sexual dysfunction (FSD) is an important but controversial problem with serious negative impact on women’s quality of life. Data from twin studies have shown a genetic contribution to the development and maintenance of FSD.

**Methodology/Principal Findings:**

We performed a genome-wide association study (GWAS) on 2.5 million single-nucleotide polymorphisms (SNPs) in 1,104 female twins (25–81 years of age) in a population-based register and phenotypic data on lifelong sexual functioning. Although none reached conventional genome-wide level of significance (10×-8), we found strongly suggestive associations with the phenotypic dimension of arousal (rs13202860, *P = *1.2×10^−7^; rs1876525, *P* = 1.2×10^−7^; and rs13209281 *P* = 8.3×10^−7^) on chromosome 6, around 500kb upstream of the locus *HTR1E* (5-hydroxytryptamine receptor 1E) locus, related to the serotonin brain pathways. We could not replicate previously reported candidate SNPs associated with FSD in the *DRD4*, *5HT2A* and *IL-1B* loci.

**Conclusions/Significance:**

We report the first GWAS of FSD symptoms in humans. This has pointed to several “risk alleles” and the implication of the serotonin and GABA pathways. Ultimately, understanding key mechanisms via this research may lead to new FSD treatments and inform clinical practice and developments in psychiatric nosology.

## Introduction

People vary in their enjoyment of sexual activity and relationships – a source of significant mental wellbeing or problems. Female sexual dysfunction (FSD) describes a cluster of sexual symptoms including desire, arousal, orgasm and pain. It appears relatively common in the general community and population-level samples and is associated with a severe decrease in quality of life in women [1]–[3]. The etiology of FSD is largely unknown although several biological and psychological correlates have been reported [3]–[5]. Nevertheless, no clear disease mechanisms have emerged and this lack of knowledge has hampered progress in both, psychiatric nosology and treatment strategies for this growing burden on women’s psychiatric health. Both, the *Diagnostic and Statistical Manual of Mental Disorders, 4th Edition* and *International Classification of Diseases, Tenth Revision* [6] have arranged FSD into categories based largely on clinical similarities, while in 1998 a consensus based definition and classification system was designed by a panel of experts during the International Consensus Development [7].

Biological research into FSD is woefully inadequate. Recent twin studies suggest FSD is familial, with genetic factors accounting for up to 51% of the phenotypic variance [8]–[10]. Twin studies also show evidence of genetic and environmental contributions to psychological factors previously linked to FSD (such as depression, anxiety, personality traits) [11]–[13]. Thus, it is possible that some of the covariation between FSD and these psychological correlates is explained by shared genetic and non-genetic factors. However, there have been no large-scale studies to identify single genes or gene variants robustly associated with FSD-phenotypes (and no genome-wide association study - GWAS - has ever been performed). To date, only a handful of candidate gene studies of sexual desire and function exist. One candidate gene study has linked serotonin polymorphisms (*5HT2A*) to reduced sexual desire as a side-effect of SSRI-medication in 89 adult men and women [14]. A further study reported an association between the dopamine D4 receptor gene (*DRD4*) with self-reports of sexual desire and arousal in 52 men and 92 women [15]. Interleukin-1beta gene (*IL-1B*) has been correlated with variation in vulvar vestibulitis syndrome scores, a broader phenotype for sexual pain symptoms [16]. All these studies have methodological shortcomings that limit their interpretation, primarily the candidate gene design, small samples in mostly clinical populations (thus lacking power to detect phenotype – DNA variant associations), and the use of non-standardized instruments that lack coverage of the phenotypic heterogeneity in sexual function. Moreover, none of these studies examine women and FSD directly, making them unsuitable for clarifying the etiological mechanisms under FSD at the population-level.

We present here the results of the first GWAS of FSD in a female population-sample published to date. By scanning a dense set of genetic variants throughout the whole genome, we can test replication of previously located genes from candidate gene investigations and also identify novel genes that may lead to the discovery of unknown biological pathways involved in the development of FSD.

## Materials and Methods

### Participants

The TwinsUK adult twin registry based at St. Thomas’ Hospital in London is a volunteer cohort of over 10,000 twins from the general population [17]. This twin population has been involved in a wide range of studies on common traits and diseases and has been shown to be representative of the general population for a wide variety of medical, behavioral, and sexual traits [3], [18], [19]. The twins were not selected on the basis of the phenotypes being studied and were unaware of any hypothesis being tested. All twins provided written informed consent and the study was approved by St. Thomas’ Hospital Research Ethics Committee.

All participants were dizygotic (DZ) pairs and monozygotic (MZ) singleton twins of white European ancestry. A total of 1,489 subjects were included, all sexually active, heterosexual with no history of any major psychological or medical condition (depression, bipolar disorder, anxiety disorder, diabetes, multiple sclerosis, endometriosis) and with all items on the Female Sexual Function Index-Lifelong (FSFI-LL) available.

### Sexual Dysfunction Phenotype

We used the recently developed 19-item Female FSFI-LL questionnaire to measure long-term variation in female sexuality, including periods of dysfunction and healthy function [20], [21]. For genetic analysis, the FSFI-LL is preferable to the “snapshot” measures used in some previous research and it better captures the variation in *enduring* female sexual functioning required for resolving the underlying genetic and non-genetic mechanisms of FSD symptoms. The FSFI-LL assesses 6 dimensions of women’s average sexual functioning “since they have been sexually active” including desire (2 items), arousal (4 items), lubrication (4 items), orgasm (3 items), satisfaction (3 items), and pain (3 items). Desire items are rated on a Likert-type scale ranging from 1 to 5. The remaining items are rated from 0 to 5 with the supplementary option “no sexual activity”. Dimension scores are derived by summing the item scores within each dimension and multiplying the sum by the dimension factor weight [20]. The dimension factor weighting converts the dimension scores to a consistent range from 0 to 6, except for the desire, which has a dimension score range from 1.2 to 6. Total scores are calculated via a simple computer algorithm and low scores on the FSFI-LL indicate more sexual problems and high scores indicate fewer problems. The FSFI-LL has excellent psychometric properties for both, total- and dimensions-specific scores, including test-retest reliability, internal consistency, external/discriminant validity [20], [21]. Exploratory and confirmatory factor analyses have successfully reproduced the original factor. According to response operator curve (ROC)-derived cut-off scores, all dimensions and the total FSD score displayed a good sensitivity to 1-specificity profile (as measured by the area under the curve = AUC), with arousal (AUC = 0.92) displaying the best trade-off and desire the lowest (AUC = 67.55%). Overall, the FSFI-LL demonstrates excellent comparability to the standard FSFI in terms of factor structure and psychometric properties [21].

Detailed information on prevalences, potential environmental risk factors and heritability estimates for FSD-symptoms in this panel are reported elsewhere (3).

### Genotyping, Quality Control, and Imputation

Genotyping was carried out using two genotyping platforms from Illumina: the HumanHap 300k Duo for a part of the TwinsUK Cohort (n = 505) and the HumanHap610-Quad array for the remainder of the TwinsUK Cohort (n = 599). Genotyping with the HumanHap 300k Duo was conducted at the Centre National de Génotypage, Duke University, NC, USA; Helsinki University, Finland; and the Wellcome Trust Sanger Institute, Cambridge, UK. Genotyping with the Infinium 610k assay (Illumina, Inc., San Diego, USA) for the remaining individuals was conducted at the Centre for Inherited Diseases Research (USA) and the Wellcome Trust Sanger Institute.

We applied stringent quality control (QC) criteria to the genotype data. Genotypes were cleaned before analysis by removing single-nucleotide polymorphisms (SNPs) or individuals not fulfilling the QC criteria. The following QC filters were applied for samples: call rate at least 95%; autosomal heterozygosity between 33 and 37%. At the SNP level, Hardy Weinberg Equilibrium (HWE) with P-value >10^−4^, Minor Allele Frequency (MAF) at least 1%, and call rate at least 95% for SNPs with MAF 0.05 and above or at least 99% for SNPs with MAF less than 0.05. We further visually inspected all intensity cluster plots of SNPs that showed either an association for over-dispersion of the clusters, biased no calling, or erroneous genotype assignment and discarded all SNPs with any of these characteristics.

Genotypes from the TwinsUK were imputed using the genotypes from the 3,855,687 autosomal markers available from the HapMap Phase 2 CEPH population [22]. After imputation using IMPUTE2 a total of 2,558,978 non-monomorphic autosomal markers became available. After removing very rare markers (MAF<0.1) and markers in Hardy-Weinberg Disequilibrium (p<e−6) and individual with poor imputation scores (<0.5), we obtained results from 2,287,762 loci across the chromosome.

### Statistical Analysis

Of the 1,489 women with recorded phenotype, genotype data was available for 1,104 subjects after QC check. Data handling and preliminary analyses were conducted using STATA software (StataCorp., College Station, TX) and Merlin (PMID 11731797) [23]. Association analyses were performed using MERLIN. Ancestry was determined through principal component analysis of individual genotypes (compared with subjects participating in the HapMap Phase II standard populations).

All traits were included in multiple regression models, with age and menopausal state included as covariates. Traits were inverse-normalized to avert undue effects of non-normality of their distributions. Regression slopes (β) are given as numbers of standard deviation units per each additional copy of the effect allele from this point onwards in the text. Given the experimental size with hundreds of thousands of SNPs being analysed individually, the commonly used “genome wide significance” (GWS) threshold was used which is the standard approximation routinely set at 5×10^−8^ [24]. However, given the cost of a strict Bonferroni adjustment in results from relatively small datasets, we considered all the associations with P≤5×10^−5^, as others have done in other studies of similar sizes (UK IBD Genetics Consortium, 2009; Amundadottir et al., 2009) [25], [26].

## Results

Racial and ethnicity stratification was checked through eigenvector analysis and the above mentioned samples only contained individuals of certified and pure European ancestry. The genotyped samples were tested for population stratification, by comparison to the three HapMap phase 2 reference populations (CEU, YRI, CHB+JPT; www.hapmap.org) using principal component analysis.

**Figure 1:**
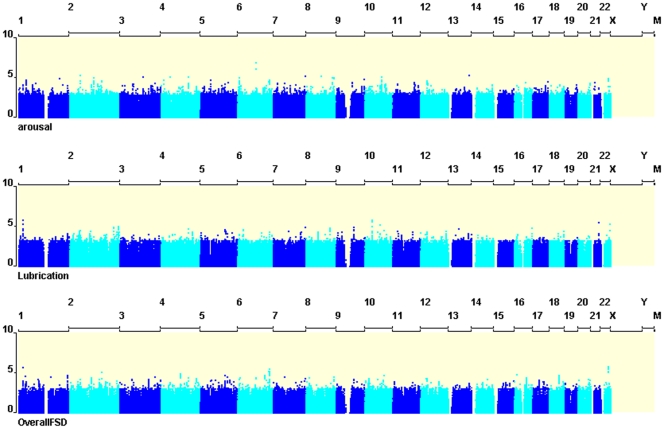
Manhattan plots describing the association of 2.5 M SNPs with sexual arousal, lubrication and overall sexual functioning. SNPs with *P*≤10–6 are highlighted with a red circle (n = 1104 females).

**Figure 2:**
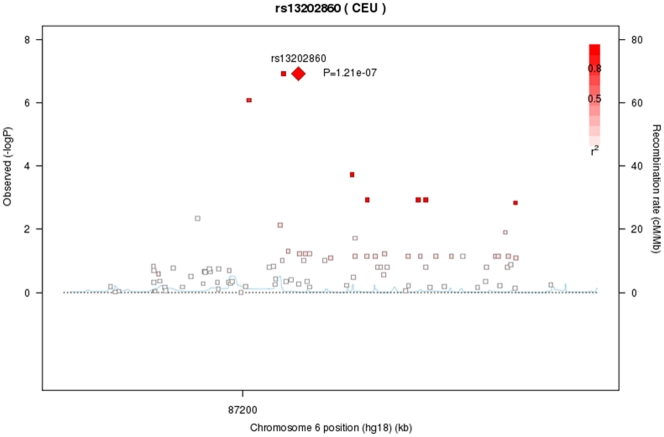
Association scatter plot for SNPs in the gene desert approximately 1Mbp upstream of the *HTR1E* gene. TwinsUK discovery cohort. Negative logarithms of the P values for the association of each SNP with spherical equivalence are plotted. The lead SNP is plotted in diamond shape, with the GWAS-analysis P value for that SNP indicated. Genotyped SNPs are plotted as squares, with the colour indicating the degree of pairwise LD between the lead and neighbouring SNPs. Red indicates strong pairwise LD, with r2≥0.8; orange indicates moderate LD, with 0.5<r2<0.8 yellow indicates weak LD, with 0.2<r2<0.5; and white indicates no LD, with r2<0.2. The recombination rates are shown as light blue line.

**Figure 3:**
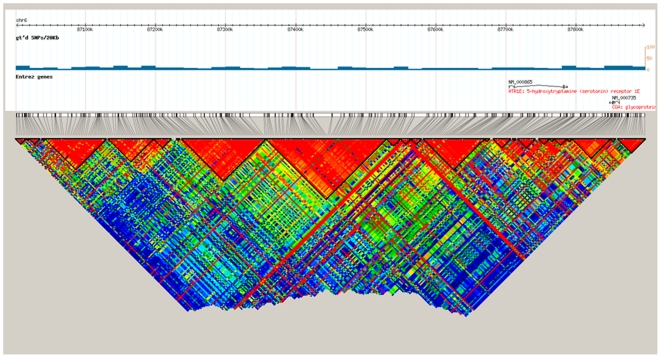
Haploview LD plot. The plot uses the hapmapPhase3 data on the CEU and the TSI Caucasian populations and encompasses an 800 kbp segment containing the associated SNP on chromosome 6 and *HTR1E*. The LD blocks were defined by confidence intervals according to Gabriel and colleagues [45]. The x-axis corresponds to the genomic position in kb and the red triangles defined by black lines represent LD blocks. The yellow arrow and the red box indicate the location of three GWAS associated SNP and the position of *HTR1E*, respectively.

The mean age of participants in the study was 57 years (range 25–81 years). The GWAS analysis was performed using both observed and imputed genotypes. The genomic inflation factor (*λ*) ranged from 0.98 to 0.99 for the different phenotypes, showing no evidence for population stratification or inflated results due to imputation. We identified 34SNPs with P-values <10^−5^ of association with FSD-related measures. These results are summarized in Table 1. The most significant association was found between rs13202860 on chromosome 6 and sexual arousal, with *P* = 1.2×10^−7^. Two additional nearby SNPs showed P-values less than 4×10^−7^, (rs1876525, rs13209281; *P* = 1.2×10^−7^ and 8.3×10^−7^, respectively; Table 1 and Figure 1). All three SNPs were associated with arousal levels and spanned a region of 11 kb, around 500 kbp upstream from the *HTR1E* (5-hydroxytryptamine receptor 1E) locus (Figure 2 and Figure 3).

**Table 1:** Top SNPs associated with sexual function related measures in a cohort of females of European ancestry.

*Phenotype*	*SNP*	*CHR*	*Position*	*Locus*	*Allele*	*Effect* *	*s.e.m*	*P*
Arousal	rs13202860	6	87211973		A	−0.421	0.08	1.213E−07
Arousal	rs1876525	6	87208811		C	−0.421	0.08	1.213E−07
Arousal	rs13209281	6	87201368		A	−0.443	0.09	8.329E−07
Overall FSD	rs4820255	22	35533796	PVALB	C	−0.366	0.076	1.687E−06
Overall FSD	rs4821535	22	35533452	PVALB	G	−0.366	0.076	1.687E−06
Overall FSD	rs2284024	22	35528729	PVALB	T	−0.366	0.077	1.838E−06
Overall FSD	rs5750311	22	35533286	PVALB	G	−0.367	0.077	2.104E−06
Overall FSD	rs739031	22	35532649	PVALB	T	−0.364	0.077	2.144E−06
Overall FSD	rs4821536	22	35533947	PVALB	T	−0.355	0.076	3.196E−06
Lubrication	rs2370759	22	32674978	EPC1	G	0.237	0.05	1.7E−06
Lubrication	rs11594963	10	32665742	EPC1	G	0.236	0.05	1.95E−06
Lubrication	rs11599044	10	32655451	EPC1	A	0.236	0.05	1.95E−06
Lubrication	rs10508773	10	32615450	EPC1	C	0.234	0.05	1.952E−06
Lubrication	rs16933243	10	32655141	EPC1	T	0.233	0.05	2.565E−06
Lubrication	rs11008865	10	32614848	EPC1	C	0.232	0.05	2.704E−06

* Effect, β coefficient of linear regression. The effect sizes denote changes in phenotype unit per each additional copy of the reference allele.

We also identified a locus on chromosome 22 with multiple adjacent SNPs showing similar, albeit modest levels of associations with overall sexual function (Table 1; Figure 1). Association was maximal for rs4820255 and rs4821535 (both *P* = 1.2×10^−6^), two SNP located 344 bp apart within intron 3 of the parvalbumin (*PVALB*) gene. Similarly, six SNPs associated with lubrication levels could be detected (*P*<3×10^−6^ for all) on chromosome 10 near the *EPC1* gene (Table 1; Figure 1).

Previous association studies have suggested several potential candidates to be associated with FSD. More specifically, earlier studies identified several variants on the dopamine D4 receptor (*DRD4*) and the serotonin 2A receptor gene (*5HT2A*) to be linked with levels of sexual desire and arousal (14,15). In this GWAS, observed and/or imputed genotype information was available for 2 SNPs in the *DRD4*, 5 SNPS in the *5HT2A* and 3 SNPS in the *IL-1B* gene, and were hence evaluated for the replication of previously reported associations. However, none of the markers showed significant associations with the previously suggested phenotypes (or with any of the outcome variables), as displayed in Table 2.

**Table 2:** P-values of available markers in our GWAS, reported to be associated with specific sexual problems in previous candidate gene studies.

Genes	SNP	Associated phenotypes	
		*P* *	*P* *
**DRD4**		**Desire**	**Arousal**
	rs3758653	0.7495	0.4242
	rs11246226	0.2303	0.7294
**HTR2A**		**Desire**	**Arousal**
	rs2760345	0.6594	0.1266
	rs7326071	0.795	0.3783
	rs2770293	0.03697	0.9446
	rs2760347	0.9225	0.1493
	rs4941570	0.04381	0.4987
**Il-B**		**Pain**	
	rs1143643	0.6775	
	rs1143634	0.7558	
	rs1143633	0.6775	

* Bonferroni corrected p-value.

## Discussion

Here we reported the results of the first ever GWAS of female sexual function levels in an unselected population-based cohort of British women. There were no associations at conventional genome-wide level of significance (P<5×10^−8^), but we found strongly suggestive associations. Several studies of similar size have considered any association with P-values ≤1×10^−5^ as being suggestive [25], [26]. Moreover, numerous suggestive associations below the genome-wide cut-off have been replicated in independent studies, strongly suggesting that these are indeed real associations rather than spurious results. For example, the Genetic Analysis of Psoriasis Consortium & the Wellcome Trust Case Control Consortium 2 in a GWAS study for psoriasis, replicated many of the suggestive associations found by Nair and colleagues including SNP nearby *IL-23A* and *TNFIP3* with *P*≤2×10^−5^ and *P*≤1×10^−5^, respectively [27], [28]. Here, we identified several strong suggestive associations with much stronger levels of significance. Our strongest association (*P* 1.2x10^−7^), was on the phenotypic dimension of arousal with a serotonin receptor gene (*HTR1E)* which represents a strong biological candidate previously shown to be involved in female sexuality. This potential susceptibility locus resides within a ∼1 Mbp segment of the genome devoid of annotated genes, located about 500 kbp downstream of the *HTR1E* gene. To assess the relationship between rs13202860 and *HTR1E* we plotted the LD pattern of the region. HapMap 3 data from two Caucasian populations (CEU and TSI) shows that rs13202860, rs1876525 and rs13209281, which show association with arousal in our study, are located in different LD blocks than *HTR1E* (Figure 3). Although *HTR1E* is an interesting candidate gene because of its known physiology, the large distance between both loci, together with the evidence that the significant SNP lie in other LD blocks than *HTR1E* clearly suggest that these three SNPs are tagging independent associations and that the causal polymorphism is more likely to regulate gene expression rather than the protein structure of *HTR1E*. Enhancers are elements of the genome that regulate gene expression of nearby or distant genes and which can be located within gene deserts [29]. Recent research suggests that polymorphisms in gene deserts could impact on disease by altering an enhancer element [29]–[31]. Thus, it is possible that our causal variant is altering an enhancer element located in the gene desert influencing *HTR1E*.

*HTR1E* encodes one of the families of highly conserved serotonin receptor genes and is strongly expressed in neurons, primarily in limbic brain regions (including caudate putamen, claustrum, hippocampus, and amygdala) [32]–[33]. This high degree of evolutionary conservation of genetic sequence suggests an important physiological role of the *HTR1E* receptor in humans. However, the actual function of the *HTR1E* receptor remains unknown. Nevertheless, *HTR1E* is a gene with considerable biological relevance to our phenotype of sexual functioning as it shares amino acid sequence homologies and some pharmacological characteristics with other 5-HT receptors (serotonin) and is therefore closely related. Comparative research has documented the critical role of serotonin receptors in modulating human and non-human mammalian sexual behavior and functioning acting on both central and peripheral (genital) sites [34]–[35]. SSRI (selective serotonin reuptake inhibitors)-associated sexual side effects, which include peripheral dysfunctions (e.g., erectile) as well as central problems in desire and arousal are well documented at high prevalence among users (up to 60%) [36]. These post-SSRI sexual dysfunctions (PSSD) point strongly to the involvement of serotonin receptors in human sexual behavior. Central serotonergic activity affects female sexual functioning via limbic projections of serotonin neurotransmitter are co-localized with norepinephrine receptors – and both transmitters seem to work in conjunction in the regulation of arousal and lubrication [35], [37]. However, the involvement of serotonin receptors in several reward-related behavioral functions (e.g. satiety, sexual behavior, nociception, escape, and stress) suggests that these receptors may function in the “higher-order” integration of rewarding behavior.

Our finding of a putative association between *PVALB* and overall sexual functioning scores on the FSFI-LL is entirely new. *PVALB* is a calcium-binding albumin protein present in GABAergic interneurons, expressed predominantly in the prefrontal cortex. Similar to serotonin, GABA is a major inhibitory neurotransmitter. Several lines of research demonstrate that GABA levels are associated with sexual function. Animal studies show that GABA(A) and GABA(B) receptors are involved in the inhibition of lordosis (a response shown by female animals indicating sexual receptivity) as well as mediating the effects of sex steroids such as estrogen in appetitive sexual behavior (e.g., sexual exploration) [37], [38]. Elevated levels of stress have also been shown to dampen sexual response in animal models as well as being a significant psychological correlate of FSD in women [39], [40]. The number of hippocampal PV-containing GABAergic interneurons is highly responsive to chronic external stressors, offering the potential of stress-induced neuro-structural alterations.

The EPC1 gene encodes the enhancer of polycomb homolog 1 and is a component of the NuA4 histone acetyltransferase complex. Previous research has suggested that this complex may be required for the activation of transcriptional programs associated with oncogene and proto-oncogene mediated growth induction, tumor suppressor mediated growth arrest and replicative senescence, and apoptosis. It has also shown to be involved in skeletal muscle differentiation [41], [42]. At this stage it is unclear through which mechanisms EPC1 could have an effect on vaginal lubrication and would need to be further investigated.

The present study had some methodological limitations and the findings should be interpreted with caution. Our study sample consisted mostly of peri- or postmenopausal women (70%). For this reason, representativeness of our study might be limited to the older female population, especially when considering that sexual dysfunction is more common in peri- and postmenopausal than in the non-climacteric period. However, prevalence rates of FSD in our sample are comparable to estimates found in other, younger populations [3]. Ideally, although similar populations are hard to come by, it would be important to replicate our GWAS findings in larger and independent samples before pursuing research into the underlying biological disease pathways. Our sample size may be one reason why our analysis did not reach conventional levels of GWA significance. Unfortunately, as is common in this field, there are no additional genotyped cohorts available with matching phenotypic data that could have been used to replicate our findings. Common diseases are typically influenced by multiple environmental as well as genetic factors. Our case and control participants may differ systematically for several of these environmental characteristics (e.g. education, anxiety levels, personality) which in turn could theoretically be related to genetic variation and to the disorder itself. Future studies in much larger sample sizes may be able to partition effects of known psychological predictors of FSD (such as sexual distress and anxiety levels) and if family-based differentiate genetic architecture of these co-morbid traits. Current approaches to perform GWAS are most successful if the common disease/common variant (CDCV) assumption holds [43]. Currently, exome sequencing has proven to be a powerful tool to identify rare coding variants and has the potential to overcome certain GWAS limitations by focusing on the identification of functional genomic structural variants rather than markers. Gene-environment interaction is also likely to have an influence on the development and maintenance of FSD [44]. In this regard, high throughput sequencing approaches would be again very useful as it can be used to interrogate functional aspects of the genome to identify epigenetic modifications such as DNA methylation, DNA-protein interaction, chromatin accessibility, etc.

In summary, we report the first GWAS of female sexual dysfunction symptoms in humans. This has pointed to several “risk alleles” and the implication of the serotonin and GABA pathways which we hope encourages further replication in large and independent population-based cohorts and then biological investigation to elucidate possible mechanisms. Ultimately, understanding key mechanisms via this research may lead to new FSD treatments and inform clinical practice and developments in psychiatric nosology.
